# Characterization of the Phosphorylation Site of GRTH/DDX25 and Protein Kinase A Binding Interface Provides Structural Basis for the Design of a Non-Hormonal Male Contraceptive

**DOI:** 10.1038/s41598-019-42857-9

**Published:** 2019-04-30

**Authors:** Murugananthkumar Raju, Sergio A. Hassan, Raghuveer Kavarthapu, Rajakumar Anbazhagan, Maria L. Dufau

**Affiliations:** 1 Section on Molecular Endocrinology, Division of Developmental Biology, Eunice Kennedy Shriver National Institute of Child Health and Human Development, OIR/CIT, National Institutes of Health, Bethesda, MD 20892-4510 USA; 2 Center for Molecular Modeling, OIR/CIT, National Institutes of Health, Bethesda, MD 20892-4510 USA

**Keywords:** Phosphoproteins, Molecular modelling

## Abstract

Gonadotropin Regulated Testicular Helicase (GRTH/DDX25), expressed in the male gonad, is essential for the completion of spermatogenesis. Our early studies revealed a missense mutation (R242H) of GRTH in 5.8% of Japanese patient population with azoospermia. Transfection of the mutant GRTH construct in COS-1 cells leads to loss of the 61 kDa cytoplasmic phospho-species. Mice with knock-in of the human GRTH mutation are sterile and lack sperm with normal androgen and mating behavior. These findings provide an avenue for the development of a non-hormonal male contraceptive. Using site directed mutagenesis and a site-specific phospho-antibody, we have identified T239, structurally adjacent to the patient’s mutant site as the GRTH phospho-site. Molecular modelling provided structural basis for the role of R242 and other critical solvent-exposed residues at the GRTH/PKA interface (E165/K240/D237), on the control of GRTH phosphorylation at T239. Single or double mutations of these residues caused marked reduction or abolition of the phospho-form. These effects can be ascribed to critical disruptions of intramolecular H-bonds at the GRTH/PKA interface, which leads to modest but consequential structural changes that can affect PKA catalytic efficiency. Inhibition of phosphorylation may be achieved by small, drug-like molecules that bind to GRTH and reconfigure the GRTH/PKA interface.

## Introduction

Gonadotropin Regulated Testicular Helicase (GRTH/DDX25) is a member of the DEAD-box family of RNA helicases identified and isolated from rat, mouse, and human testes^1,2^. This testis-specific protein expressed in Leydig and germ cells of the male gonad is transcriptionally upregulated by gonadotropin through direct and indirect actions of androgen via androgen receptor, respectively^3–5^. In Leydig cells, GRTH regulates cholesterol homeostasis via its action on StAR expression^6^. In germ cells, it is expressed predominantly in meiotic spermatocytes and round spermatids where is essential for spermatid development and completion of spermatogenesis. GRTH knockout mice lack sperm due to failure of round spermatids to elongate at step 8 of spermiogenesis^7^. This multifunctional protein participates in the export of relevant mRNAs from nucleus to cytoplasmic sites of germ cells for storage/degradation in chromatoid bodies of round spermatids and translation, as indicated through its association with actively translating polyribosomes in germ cells^8,9^. It was also found to be a regulator of miRNA biogenesis^10^ and an inhibitor of germ cell apoptosis^11^.

In germ cells of mouse and rat, there are two species of GRTH, a 56 kDa non-phosphorylated form, predominantly found in the nucleus, where it interacts with CREM and participates in mRNA transport, and the 61 kDa phosphorylated form (pGRTH), present exclusively in the cytosol and found to be associated with polyribosomes. The non-phospho form was also found in the cytoplasm of germ cells. In COS-1 cells transfected with GRTH cDNA, a similar pattern of expression of these forms was observed^8^. GRTH phosphorylation was induced by cAMP in COS-1 cells transfected with GRTH. The phospho-species was significantly increased with overexpression of Protein Kinase A catalytic subunit (PKA-Cα) and prevented by co-expression of rabbit protein kinase inhibitor (PKI). The 61-kDa species was detected in Western blot by phospho-Thr antibody but not by Ser or Tyr antiserum^8^.

Our previous studies revealed the presence of a missense mutation of arginine to histidine at position 242 (R242H) in exon 8 of GRTH in 5.8% of Japanese patients with azoospermia^12^. Transfection of this mutant in COS-1 cells causes loss of the 61 kDa cytoplasmic phospho- species with preservation of the non-phospho GRTH form^12^. Recently, a knock-in (KI) model created in our laboratory based on the R242 to H mutant revealed the functional relevance of this mutation. KI mice are sterile with reduction of testicular size, lacking sperm due to arrest at step 8 of round spermatids and complete loss of the pGRTH with unchanged non-phospho form. The lack of pGRTH does not affect the levels of plasma testosterone or mating behavior but solely inhibits sperm formation^13^.

In this study, using site directed mutagenesis, we identified the GRTH/DDX25 phosphorylation site and the critical residues at the GRTH/PKA interface that control the phosphorylation status of the protein. Changes in basicity in the environment of position 242 upon R to H mutation or local conformational changes induced by this structural perturbation can affect phosphorylation by PKA at an adjacent putative site. Molecular modeling provided a structural rationale for these effects and guided the selection of residues targeted for mutations that abolish GRTH phosphorylation. The partial mapping of the phospho-site and the GRTH/PKA interface allowed us to identify critical structural motifs that are necessary for cAMP/PKA binding and/or catalysis; local perturbation of these motifs can lead to complete abolition of phosphorylation, hence spermatogenic arrest. Collectively, these findings provide an avenue for the development of a male contraceptive through compounds that mimic the human mutation or that directly or indirectly block the action of PKA on the phosphorylation site selectively and specifically.

## Results

**Figure 1:**
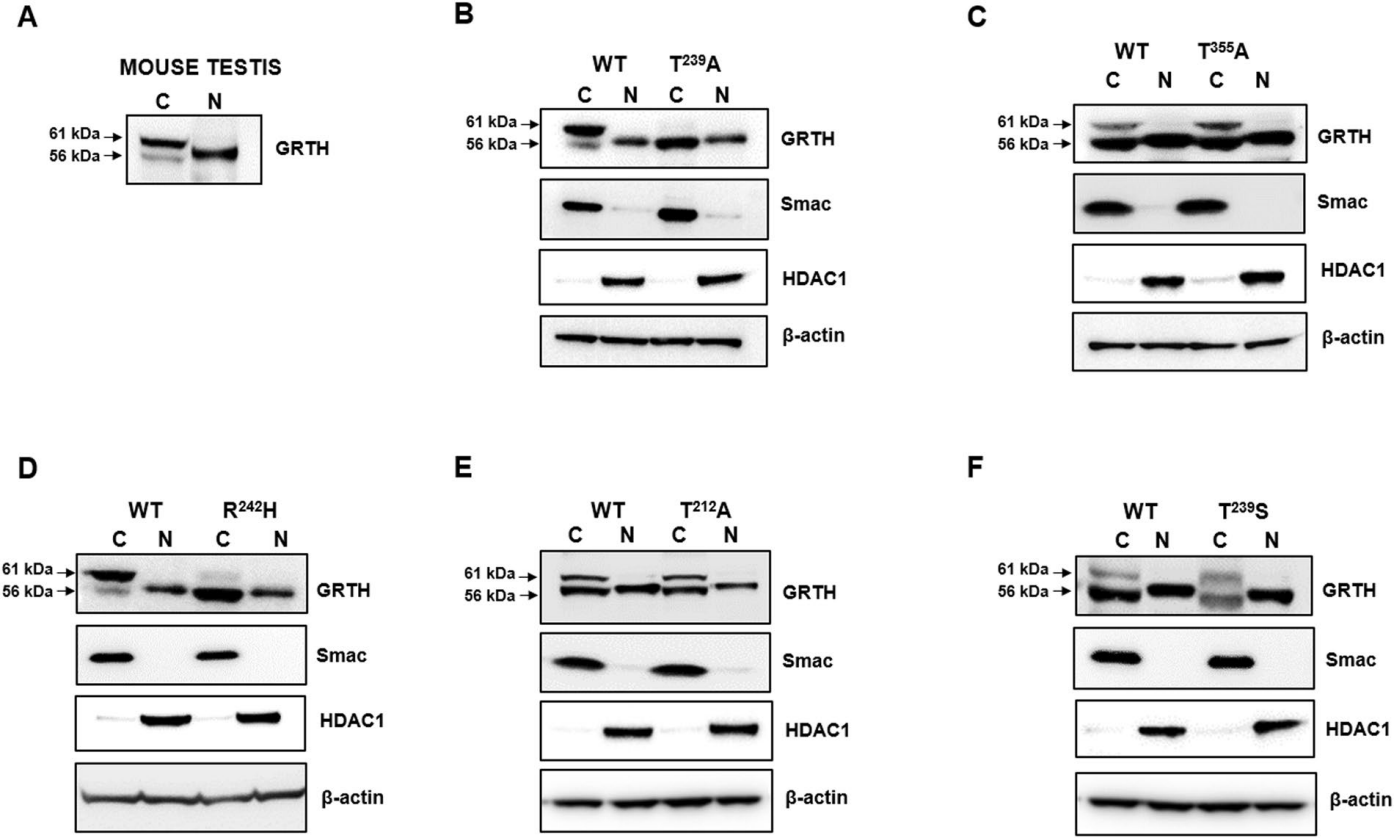
Determination of the phosphorylation site of GRTH. Expression of hGRTH wild-type and mutant proteins in COS-1 cells. (**A**) Western blot analysis of cytoplasmic and nuclear extract of mouse testis was performed to validate the expression of pGRTH (~61 kDa) and non-pGRTH (~56 kDa) forms in the cytoplasmic fraction and non-pGRTH form in the nuclear fraction (**C**- Cytoplasmic extract; N- Nuclear extract) as a positive control to be compared with GRTH mutants T239A (**B**), T355A (**C**), R242H (**D**), T212A (**E**), T239S (**F**). GRTH protein expression in C and N fractions of COS-1 cells. Nuclear HDAC1 and mitochondrial Smac proteins were assessed to check the efficient separation of N and C extracts, respectively; β-actin was used as internal control. A minor difference in migration of non-phospho GRTH band in nuclear fraction compared to cytosolic fraction is noted. Full-length blots are presented in Supplementary Figs S1, S2 and S3.

Based on our initial studies indicating that the cytoplasmic form of GRTH was phosphorylated at a threonine residue^8^, scanning analysis of consensus PKAα phosphorylation motifs identified five putative phosphorylation sites at positions 38, 212, 239, 355 and 408^12^. Site directed mutagenesis and expression of the corresponding constructs in COS-1 demonstrated that the mutation T239A and R242H completely abolished the 61 kDa phospho-band with preservation of the non-phospho species when compared with wild-type construct (Fig. 1B,D). Only the non-phospho species was found in the nucleus and cytoplasm of the cells. This resembles the endogenous expression of non-phospho GRTH in germ cells of mouse testis (Fig. 1A). In contrast, mutations T355A or T212A had no effects (Fig. 1C,E), whereas effective phosphorylation was retained in the T239S mutant (Fig. 1F). Thus, the site of phosphorylation was unambiguously identified at position 239, which is upstream in the sequence and structurally closed (see below) to the R242H mutation in infertile patients. The PKA recognition sequence (TKIR) is unusual as a canonical consensus motif^14^, but recent studies of tissue-specific phospho-sites pS/pT indicate that (pS-X-X-R) is common in testis^15^. These findings lay the foundation for further probing and structural analysis of the GRTH/PKA binding interface with the goal to identify potential binding sites for small drug-like molecules that could elicit similar deleterious effect on T239 phosphorylation as does H242.

**Figure 2:**
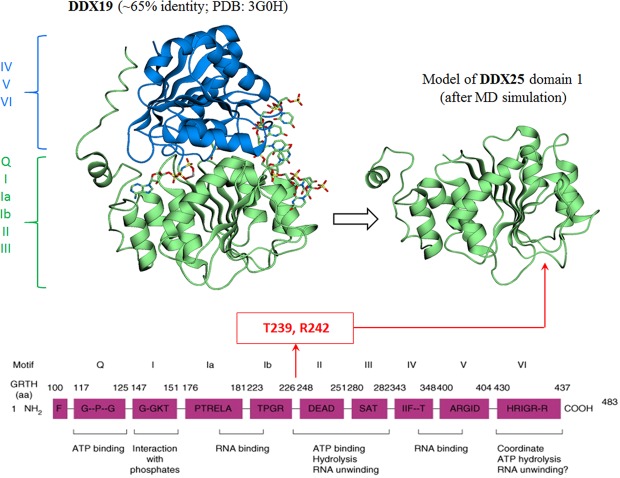
Modeling of GRTH/DDX25. Left panel: Crystal structure of the DDX19 helicase^24^ (PDB: 3G0H) with the domain 1 (green) and 2 (blue) indicated; the co-crystalized fragment of RNA and an ATP-analog are also shown. Right panel: snapshot at the end of a MD simulation of the model of domain 1 of DDX25 obtained by threading the corresponding sequence on the structure of domain 1 of DDX19. The location of the phosphorylation site at T239 and of the critical residue R242 are shown in the structure (this study) and in the sequence^2^.

**Figure 3:**
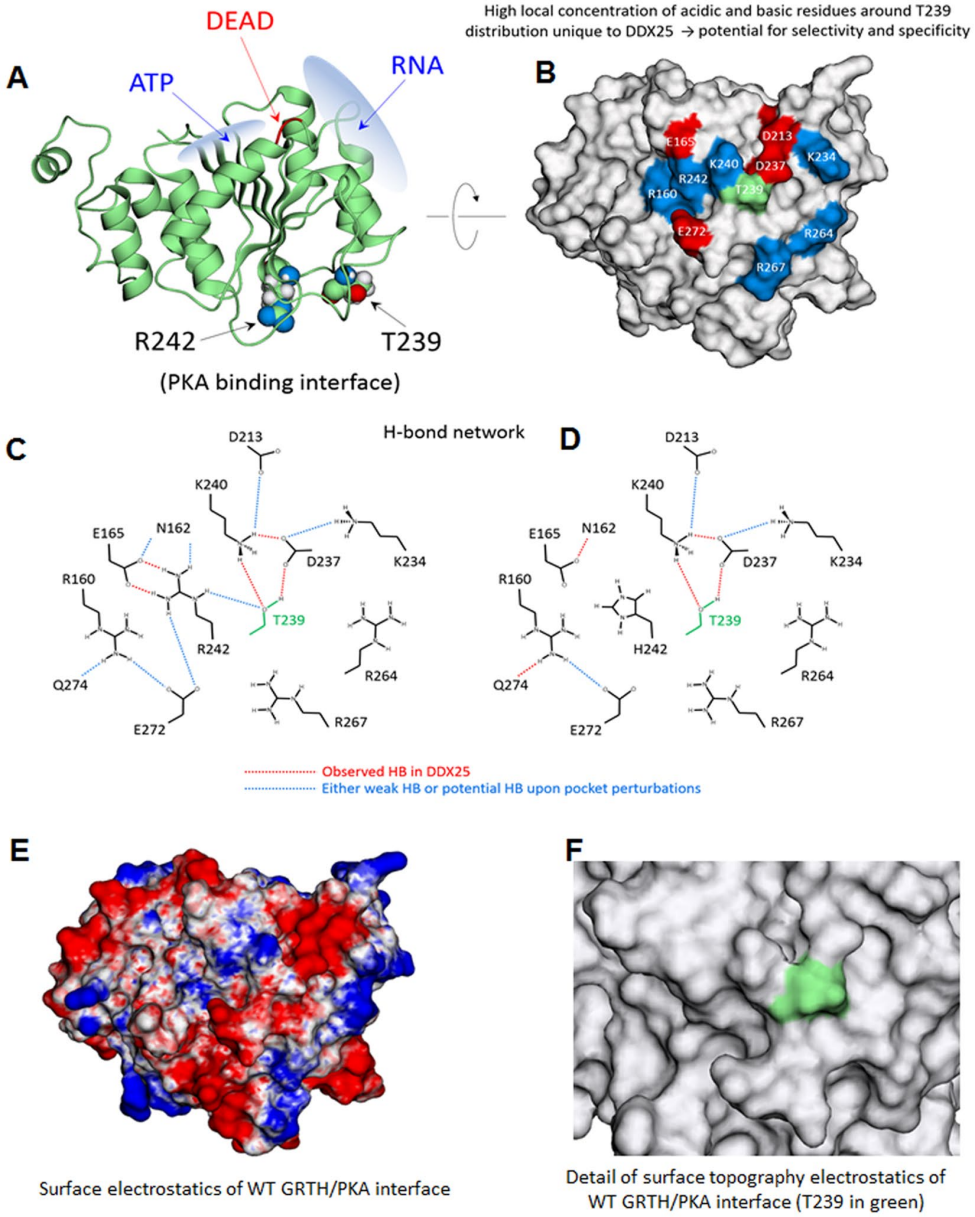
Structural characterization of GRTH. (**A**) Domain 1 of DDX25 showing the binding sites of ATP and RNA along with the DEAD-box sequence and the relative positions of T239 and R242. Both residues are solvent exposed and located at the GRTH/PKA binding interface. (**B**) GRTH/PKA interface on GRTH indicating the basic (blue) and acidic (red) residues surrounding T239; most of these residues are unique to DDX25 including T239 itself; only 5 out of the 12 residues shown are common between DDX25 and DDX19. (**C**) Intramolecular H-bond network of residues at the GRTH/PKA interface for wild-type GRTH. The network was identified from MD simulations and deemed statistically significant in frequency: red lines indicate strong/persistent H-bonds; blue lines are either weak bonds or strong bonds developed upon mutations of interfacial residues; simulations were carried out for single mutants E165A, K234A, K234D, K240A, K240D, D237H, D237A, R242H (neutral and protonated histidine), R242A and for the double mutants E165A + E272A, E165A + R242A, E165A + K240A. The MD results were used to suggest mutations to be carried out experimentally. (**D**) like in (**C**) but for H242 (protonated) observed in infertile patients^12^. (**E**) Surface electrostatic potential on GRTH at the GRTH/PKA interface; same orientation as in (**B**) (red: negative field, blue: positive field, white: neutral field; drawn at the same magnitude of positive and negative field intensities). Note the negative elongated crevice flanked by two regions of positive field; hydrophobic regions are generally outside the interface. (**F**) Details of surface topography at the interface with T239 highlighted in green.

GRTH/DDX25 contains 483 residues and share all nine conserved motifs of the DEAD-box family of RNA helicases (Q, I, Ia, Ib, II, III, IV, V, VI)^1,2,8,16–18^. The crystal structure of DDX25 has not been resolved, so it was here modeled based on the structure of DDX19, the member of the family with the closest amino acid sequence identity (~65%) to DDX25 (Fig. 2). DEAD-box helicases contain two globular domains, 1 and 2, which comprise the conserved regions (Q, I, Ia, Ib, II, III) and (IV, V, VI), respectively (Fig. 2). Since the phosphorylation site T239 and the critical residue R242 both reside in the region between the conserved sequences Ib and II, modeling was concentrated on domain 1. Figure 3(A,B) shows the wild-type protein with T239 and R242 and the distribution of basic and acidic residues at the GRTH/PKA interface. These residues are distant from the binding sites of both RNA and ATP molecules and do not directly interact with the DEAD-box sequence. All these residues are solvent-exposed and engaged in a complex network of intramolecular H-bond interactions (Fig. 3C), perturbations of which have the potential to abrogate phosphorylation either by directly affecting PKA binding or catalytic activity.

**Figure 4:**
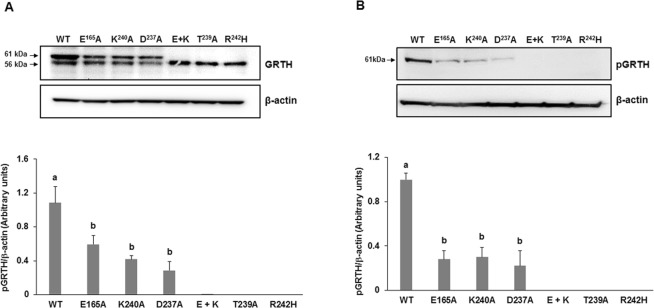
Effects of single and double mutations on GRTH phosphorylation. Western blot analyses of pGRTH wild type and mutants (E165A, K240A, and D237A, E165A + K240A, T239A and R242H) expression. Cytoplasmic protein extracts prepared after transfection of COS-1 cells with GRTH wild type and mutants were subjected to Western blot analysis. Blots were incubated with peptide GRTH antibody (amino acid 465–477) purified by protein A-Sepharose (**A**) which recognize pGRTH (61 kDa) and the non-phospho 56 kDa species, or with a site-specific phospho-peptide GRTH antibody (**B**). β-actin was used as an internal control. The protein band intensities of pGRTH (61 kDa) from three independent experiments (mean ± SEM) were measured using ImageJ software and normalized with the β-actin. Different superscript letters (a and b) indicate significant differences (*P* < 0.05, One-way ANOVA with Tukey’s multiple comparison test). Full-length blots are presented in Supplementary Fig. S4.

Residue E165 interacts with R242 through two persistent H-bonds; this strong interaction appears to be critical for GRTH phosphorylation since disengagement of R242 by a single E165A mutation highly reduced T239 phosphorylation. Changes in the cytosolic form of GRTH with preservation of the non-phospho form are shown in Western Blots of cytoplasmic extracts of COS-1 cells transfected with wild-type and mutants GRTH using a polyclonal antibody to GRTH (Fig. 4). Likewise, direct mutation of R242H weakens the strong H-bond interaction with E165 leading to persistent exposure of the histidine side chain to the solvent (Fig. 3D). This induces changes in both surface electrostatics and H-bond pattern, which may directly affect PKA binding affinity or introduce local perturbations to indirectly affect transfer of the phosphoryl group to the nearby T239. These perturbations include enhanced/reduced flexibility of the loop containing the PKA recognition motif (TKIR) due to re-organization of intramolecular H-bonds. The strong double H-bond between R242 and E165 in the wild-type protein can also be disrupted by a single E165A. In this case, the side chain of R242, unlike H242, rearranges itself to find other intramolecular H-bond partners (most commonly the carbonyl group of R160), which perturbs the PKA binding site in yet another manner. This single E165A mutation also causes K240 to disengage from T239 and more directly interact with PKA, which may be deleterious to phosphorylation. Similar changes in H-bond pattern and surface electrostatics occur upon mutation of other solvent-exposed residues, including K240 and D237, both of which greatly impairs phosphorylation at T239 (Fig. 3E,F). Major changes in H-bond pattern were observed in the double mutant E165A and K240A, which completely abolished phosphorylation, mimicking the effects of the naturally occurring R242H. These findings were further confirmed using a site-specific phospho-antibody (pT239). The 61 kDa phospho-species was reduced in single mutants E165A, K240A or D237A, and are not detected in the double mutant E165A and K240A, as is the case of patients with the single R242H mutation and in the T239A mutation of the phospho-site (Fig. 4A,B).

**Figure 5:**
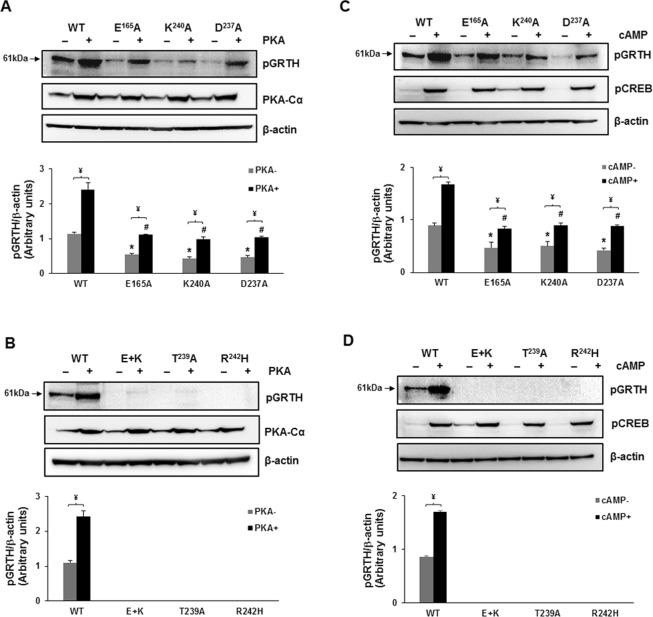
Effect of PKA and cAMP on the expression of GRTH wild type and mutants that caused reduction or abolition of pGRTH at T239. (**A** and **B**) Western Blot analyses of GRTH and mutants co-expressed in COS-1 cells with or without PKA-Cα construct or empty vector. Western Blots of cytoplasmic cell fraction were probed with site-specific GRTH phospho-antibody and PKA-Cα antibody. (**C** and **D**) Western blot of the cytoplasmic fraction prepared from cells transfected with GRTH wild type and mutants and incubated with or without 500 mM cAMP for 24 h were probed with site-specific GRTH phospho-antibody. Expression of phospho-CREB was shown as positive control indicating cAMP activity. The protein band intensities of pGRTH from three independent experiments (mean ± SEM), measured using ImageJ software were normalized by β-actin. ¥ indicates significant difference between with or without PKA and cAMP groups. * and # indicates significant reduction in the expression of pGRTH compared to WT group (*P* < 0.05, One-way ANOVA with Tukey’s multiple comparison test). Full-length blots are presented in Supplementary Figs S5 and S6.

Co-expression in COS-1 cells of the catalytic subunit of PKA with either wild-type GRTH or with the mutants relevant for phosphorylation by endogenous PKA, revealed that overexpression of PKA-Cα minimally relieved the inhibition of phosphorylation caused by E165A, K240A and D237A to only a small fraction when compared to its effect for wild-type GRTH (Fig. 5A,B). In contrast, it was ineffective when phosphorylation was completely abolished by either the double mutation E165A + K240A or by R242H, indicating a central role of these residues in the structural integrity of the site, either for PKA binding or for the efficiency in the phosphoryl transfer. The lack of pGRTH with the T239A mutation further confirm the sole participation of T239 in GRTH phosphorylation. When the endogenous enzyme was activated by the addition of cAMP to the cultures, changes were like those observed with overexpression of the active enzyme (Fig. 5C,D).

**Figure 6:**
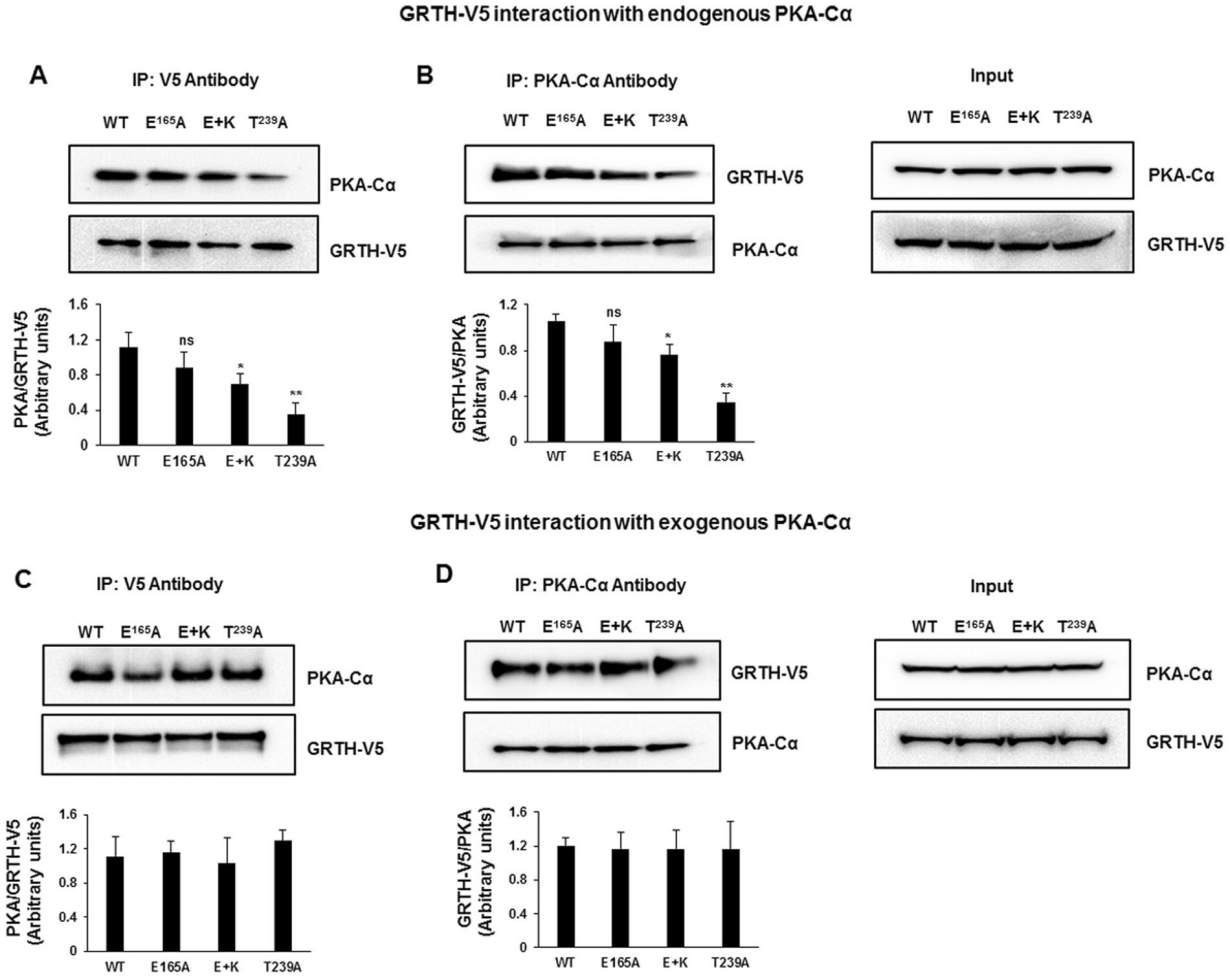
Interaction of PKA-Cα with GRTH-WT and mutants (E, E + K and T239). COS-1 cells expressing GRTH-V5 were IP with V5 antibody (**A**) and PKA-Cα antibody (**B**) and subjected to western blots developed with both V5 and PKA antibodies. Input showing endogenous PKA and overexpressed GRTH-V5 (Right). COS-1 cells co-transfected with GRTH-V5 and PKA-Cα were IP with V5 antibody (**C**) and PKA-Cα antibody (**D**) and subjected to western blots developed with both V5 and PKA antibodies. Input showing endogenous overexpressed PKA and GRTH-V5 (Right). Protein band intensities were quantified using ImageJ software. Asterisk indicate significant difference compared to WT (*P < 0.05, **P < 0.01, ns – not significant; One-way ANOVA with Tukey’s multiple comparison test). Full-length blots are presented in Supplementary Fig. S7.

Subsequently, the association of PKA-Cα with GRTH and mutants was examined using Immuno-Precipitation (IP) of COS-1 cells extracts that were transfected with the GRTH-V5 construct alone or co-transfected with the PKA-Cα using either V5 and PKA-Cα antibodies. We observed co-IP of GRTH and PKA-Cα using either of these antibodies (Fig. 6A–D). The interaction of endogenous PKA with GRTH mutants (E + K and T239) was reduced compared to GRTH-WT (Fig. 6A,B). However, when PKA-Cα was overexpressed there was no significant change in association of PKA-Cα with GRTH-WT and GRTH mutants (E, E + K and T239; Fig. 6C,D). These results indicate that the PKA-Cα association with the mutants was not impaired (for overexpressed PKA-Cα) or only mildly reduced (endogenous) when compared with WT-GRTH or with the single E165A mutation. In contrast, it is the efficiency of the catalytic activity of PKA-Cα that appears to be completely abolished in E + K and T239 mutants [See Fig. 5]. This demonstrates that mutations at the GRTH/PKA interface lead, at least for the mutations tested, to atomic reconfigurations that are modest enough to still allow binding of the kinase but strong enough to prevent phosphorylation by a constitutively active catalytic subunit. This lends support to the notion that binding of a small, drug-like molecule at the interface needs only perturb the atomic arrangement in the environment of T239 to dramatically inhibit the enzyme activity.

## Discussion

In this study we have identified residue T239 as the phosphorylation site of GRTH. Mutation of T239 abolished the pGRTH cytoplasmic 61 kDa species, whereas the non-phospho 56 kDa form was present in the nucleus and in the cytoplasm of transfected COS-1 cells. This was further confirmed with a GRTH peptide antiserum and by a highly site-specific phospho-peptide antibody. The GRTH phosphorylation sequence ^239^TKIR conforms to the PKA recognition motif pS-X-X-R more common in testis than in other tissues^15^. Phosphorylation of GRTH was also observed in the mutant S239, although it was found to be less efficient.

There is growing evidence that post-transcriptional modifications, including phosphorylation, of members of the DDX family regulate protein function. These modifications are mostly present in the divergent flanking sequences (N- and C-terminal), presumably with specific inherent functions; some of these, however, are also present in core region^19^. GRTH/DDX25 phosphorylation is relegated exclusively to T239 in the core region and not within any of the conserved domains of the DDX family. The GRTH phospho-species is essential for the cytoplasmic functions of this helicase, including shuttling of messages to the chromatoid body and polyribosomes, maintenance of the structure of the chromatoid body of the round spermatid, and the expression of messages relevant for completion spermatogenesis^8,13,20^. Moreover, the phospho-species differs from the non-phospho form whose functions are concerned with the transport of messages from nuclear to cytoplasmic sites through a CREM related mechanism. The phospho-site of GRTH resides in the structure adjacent to R242 found as a heterozygous missense mutation to histidine in a Japanese population of infertile men^12^. This mutation abolishes phosphorylation of T239, and we have also identified in this study other single and double mutants, not within the PKA consensus sequence, that are essential for phosphorylation. Molecular modelling based on the crystal structure of the RecA domain 1 of DDX19, which shares overall 65% amino acid identity with GRTH, has identified relevant structural motifs on the GRTH surface required for productive association with PKA. The binding site is rich in basic and acidic residues that engage in a complex network of intramolecular H-bonds unique to DDX25. The loop containing the PKA-targeting motif is amid such arrangement and then susceptible to structural changes of the local environment. The mutual proximity of several charged/H-bond-prone residues renders the binding pocket unstable, leading to topographic reconfiguration upon local perturbations with significant inhibition or abolition of phosphorylation by PKA.

Molecular modeling and mutagenesis analysis led us to conclude that disruption of strong intramolecular H-bonds (between either, E165 and R242 or between K240 and D237) is the reason for the major reduction of T239 phosphorylation by single mutations (E165A or K240A). Complete abolition was achieved only by the double mutation (E165A and K240A). Overexpression of the PKA catalytic subunit (PKAc) or its endogenous generation/activation by cAMP partially relieved the reduction induced by the single mutations but was ineffective in the case of the double mutants, in which case complete abolition was retained (Fig. 5). The present studies have indicated that the deleterious effects induced by the mutations (here demonstrated for E + K) on the phosphorylation of GRTH for the most part do not stem from a decrement of the PKA-Cα binding affinity for GRTH but it is mostly related to specific changes in the delicate atomic arrangement of the substrate pocket necessary for catalytic efficiency^21^.

Blocking phosphorylation of T239 in GRTH/DDX25 with a small chemical compound should thus provide an effective (selective and specific) oral male contraceptive. The results of this study suggest that a small, drug-like molecule that bind to GRTH at the GRTH/PKA interface could trigger modest by consequential local perturbations with similar effects to those revealed by mutations *in vitro* (this study) and *in vivo*, leading to abrogation of spermatogenesis in mice^13^. The local topography of GRTH which is relatively shallow with no deep crevices (Fig. 3F), may at first sight appear unattractive as a binding site for a high-affinity, non-toxic drug. However, the unique distribution of basic/acidic surface residues, its sensitivity for perturbations of the pocket that abrogate formation of pGRTH, and the recognition of the site by PKA to effect competent phosphorylation provide strong impetus for development of an effective pharmacophore^22^. Virtual and experimental throughput screening using stable COS-1 cell lines expressing GRTH in combination with our specific pGRTH antiserum should be applicable to the various available current methodologies that could lead to the discovery of a reversible non-toxic oral male contraceptive drug.

## Materials and Methods

### Mutagenesis

Point mutations of human GRTH-plasmids were prepared using the QuikChange II Site-Directed Mutagenesis Kit (Agilent technologies) as per the protocol. PCR reaction was setup using mutagenesis-grade *PfuUltra* HF DNA polymerase with the oligonucleotide primers containing the required point mutation (see Table 1). The PCR product was treated with DpnI supplied with the kit to digest the methylated parental DNA template. The nicked vector plasmid DNA containing the chosen mutation was then transformed into XL1-Blue cells. The mutated plasmid was selected, confirmed by sequencing, and transfected into COS-1 cells for expression.

**Table 1:** List of primers used for Site-Directed Mutagenesis.

Mutant	Primers name	Nucleotide sequence (5′ ---- 3′)
R^242^H	GRTH R242H Fw	TTGACTAAGATTC*A*TGTGTTTGTCCTG
	GRTH R242H Rv	CAGGACAAACACA*T*GAATCTTAGTCAA
T^239^A	GRTH T239A Fw	GATTGATTTG*G*CTAAGATTCGTGTGTTTGTCCTGG
	GRTH T239A Rv	CCAGGACAAACACACGAATCTTAG*C*CAAATCAATC
T^212^A	GRTH T212A Fw	CGAATTCCCAGAGGC*G*CCGACATCACTAAACAG
	GRTH T212A Rv	CTGTTTAGTGATGTCGG*C*GCCTCTGGGAATTCG
T^355^A	GRTH T355A Fw	CGCTAAGTGGTTG*G*CCGTGGAGATGATACAG
	GRTH T355A Rv	CTGTATCATCTCCACGG*C*CAACCACTTAGCG
T^239^S	GRTH T239S Fw	GATTGATTTGA*G*TAAGATTCGTGTGTTTGTC
	GRTH T239S Rv	GACAAACACACGAATCTTA*C*TCAAATCAATC
E^165^A	GRTH E165A Fw	GTTAATGCCTTGG*C*ATTGTTCCCACAGTGC
	GRTH E165A Rv	GCACTGTGGGAACAAT*G*CCAAGGCATTAAC
K^240^A	GRTH K240A Fw	GATTGATTTGACT*GC*GATTCGTGTGTTTGTC
	GRTH K240A Rv	GACAAACACACGAATC*GC*AGTCAAATCAATC
D^237^A	GRTH D237A Fw	CTAAAATTGATTG*C*TTTGACTAAGATTCGTG
	GRTH D237A Rv	CACGAATCTTAGTCAAA*G*CAATCAATTTTAG

### Transient transfection of GRTH cDNA into COS-1 cells

The full-length human GRTH cDNA (pGRTH-SPORT; GenBank Acc # AF155140) was used in the present study^1,3^. The plasmid DNA was sequenced and confirmed by the dideoxy-nucleotides chain termination method. COS-1 (ATCC^®^ CRL-1650™) cells were cultured in T75 flask at 37 °C with 5% CO_2_ containing Dulbecco Modified Eagle Medium (DMEM) high glucose, GlutaMax^TM^ Supplement, HEPES (#10564011, Thermo Fisher Scientific) supplemented with 10% fetal bovine serum and 1X Antibiotic-Antimycotic (#15240062, Thermo Fisher Scientific). Human pGRTH-Sport constructs of wild type (WT) and mutants (see Table 1) were transfected into COS-1 cells with Lipofectamine reagent (Invitrogen). The cells were incubated further for 24 h before harvesting for western blot analysis.

### Analysis of phosphomodification of GRTH in normal and mutant expression

To evaluate the effect of protein kinase A (PKA) on phosphorylation of GRTH-WT and mutants, COS-1 cells were transfected with plasmids (15 μg) expressing full-length GRTH-WT and mutants (E165A, K240A, D237A, E165A + K240A [double mutant], T239A, and R242H) alone or co-transfected with plasmid (15 µg) expressing the PKA α catalytic subunit (PKAα) in 10 cm culture dish and empty plasmid was used for equalization, and cultured further for 24 hr after transfection. Cytoplasmic protein was prepared as described below for analysis by Western Blots. In other studies, 8-bromo, 0.05 µM (Sigma, Aldrich), was added to COS-1 cells transfected with full length GRTH cDNA and further incubated for 24 h at 37 °C.

### Western blot analysis

Nuclear and cytoplasmic protein extracts were prepared from COS-1 cells using NE-PER™ Nuclear and Cytoplasmic Extraction Reagents (#78833; Thermo Fisher Scientific, Waltham, MA, USA), containing 1 × protease and phosphatase inhibitor cocktail (Thermo Fisher Scientific) following the manufacturer’s protocol. Cytoplasmic extraction reagents (CER I and II) were added to the cell pellet and centrifuged at ~16,000 × g to obtain the supernatant (cytoplasmic fraction) leaving the insoluble pellet (nuclear fraction) which was then suspended in ice-cold nuclear extraction reagent (NER). The pellet was vortex for 15 secs for every 10 mins, for a total of 40 mins to extract the nuclear fraction. Concentration of protein for each extract was determined using Quick Start™ Bradford Protein Assay (#5000201; Bio-Rad). Protein (30 µg) separated by 4–12% Bis-Tris Protein Gels was transferred to nitrocellulose membranes and incubated with a specific affinity-purified anti-GRTH rabbit polyclonal antibody^1^ or custom made affinity purified phospho-site-specific GRTH polyclonal antibody raised in rabbit to the peptide sequence (CKLIDL[pT239]KIRV) of GRTH (1:2000). Goat anti-Rabbit IgG (H + L) Poly-HRP (1:5000) was used as the secondary antibody and the immunosignals were detected by the FluorChem E system (Protein simple, CA, USA). PKA and pCREB was detected using PKA-Cα and pCREB specific antibodies (Cell Signaling CA), respectively. The 61 kDa pGRTH band intensity was measured using ImageJ software and normalized with β-actin.

### Immunoprecipitation (IP)

COS-1 cells were transfected with GRTH-V5-His construct alone or co-transfected with the PKA-Cα construct, were lysed using RIPA lysis buffer containing halt protease and phosphatase inhibitor cocktail (ThermoScientific). Total lysates (0.5 mg) were initially subjected to preclearing by incubation with 50 μl of protein A/G-agarose beads and 1 μg of rabbit IgG in IP binding buffer (ThermoScientific) with gentle agitation for 30 mins at 4 °C. Then the supernatant was incubated with either 4 μg of V5 antibody or PKA-Cα antibody overnight at 4 °C to co-immunoprecipitate PKA-Cα or GRTH-V5, respectively. Protein A-agarose beads (50 μl) was added and incubated for 4 h at 4 °C. The IP complex bound to protein A-agarose was washed four times with IP binding buffer and later eluted from beads using 1x LDS sample buffer (ThermoScientific) at 100 °C for 5 mins. The eluted samples were subjected to Western blot analyses for detection GRTH using their respective antibodies.

### Molecular modeling

The structure of DDX25 was modeled by threading its amino acid sequence on the structure of DDX19, which was co-crystalized with an ATP analog and a short RNA fragment (PDB ID: 3G0H)^23,24^. DDX19 shares about 65% sequence identity with DDX25^25^. Only the domain 1, which contains the DEAD-box and R242 was considered, comprising a total of 233 residues (H80 through L302). Upon removal of ATP and RNA, the wild-type DDX25 protein was subjected to a 20-ns molecular dynamics (MD) simulation to obtain a relaxed conformation, which was taken as the initial structure for all subsequent simulations. Single or double mutants (Table 1) were created by directly replacing the corresponding amino acid(s) on the relaxed DDX25 structure. Standard protonation states (pH 7) were used for all residues; histidine residues were unprotonated, although both neutral and protonated H242 were considered to probe its local effects on surface electrostatics and H-bond patterns. Under these conditions the wild-type protein had a total of 48 charged residues and no net charge. When the mutant led to a charge imbalance, either Na^+^ or Cl^−^ were placed randomly in the liquid phase to neutralize the system; no additional ions were added. Simulations were carried out with the all-atoms (param22) CHARMM force field (c42 version) with PMAP corrections^26^ in a cubic cell of ~9 nm of side length, filled with TIP3P water, at constant temperature (35 °C) and pressure (1 atm). Cubic PMC and PME were for the treatment of electrostatics. (For additional details of the simulation setup, see^27^ and references therein). Upon gradual heating and equilibration, the simulations were extended up to 50 ns and the last 30 ns were used to collect sufficient statistics for analysis.

### Statistical Analysis

Data are presented as the mean ± SEM of three independent experiments. Mean values of the data was compared and analyzed by one-way analysis of variance (ANOVA) followed by Tukey’s multiple comparison test using the Prism software program (GraphPad Prism 7.02 Software, San Diego, CA). A probability of P < 0.05 was considered statistically significant.

## Supplementary information

Supplimentary Figures [Full Blots]
