# Supplementary material for: Characterization of the Phosphorylation Site of GRTH/DDX25 and Protein Kinase A Binding Interface Provides Structural Basis for the Design of a Non-Hormonal Male Contraceptive
Source: Sci Rep. 2019 Apr 30;9:6705. doi: 10.1038/s41598-019-42857-9 (PMC6491591; doi:10.1038/s41598-019-42857-9)
Supplement: Supplementary file 1 — Supplimentary Figures [Full Blots] [file 41598_2019_42857_MOESM1_ESM.pdf]

Characterization of the Phosphorylation Site of GRTH/DDX25 and Protein Kinase A Binding Interface Provides Structural Basis for the Design of a Non-Hormonal Male Contraceptive

Murugananthkumar Raju[1,a], Sergio A. Hassan[2,a], Raghuveer Kavarthapu[1], Rajakumar Anbazhagan[1] and Maria L. Dufau[1*]

[1] Section on Molecular Endocrinology, Division of Developmental Biology, Eunice Kennedy Shriver National Institute of Child Health and Human Development; [2] Center for Molecular Modeling, OIR/CIT, National Institutes of Health, Bethesda, MD 20892-4510

Fig. S1

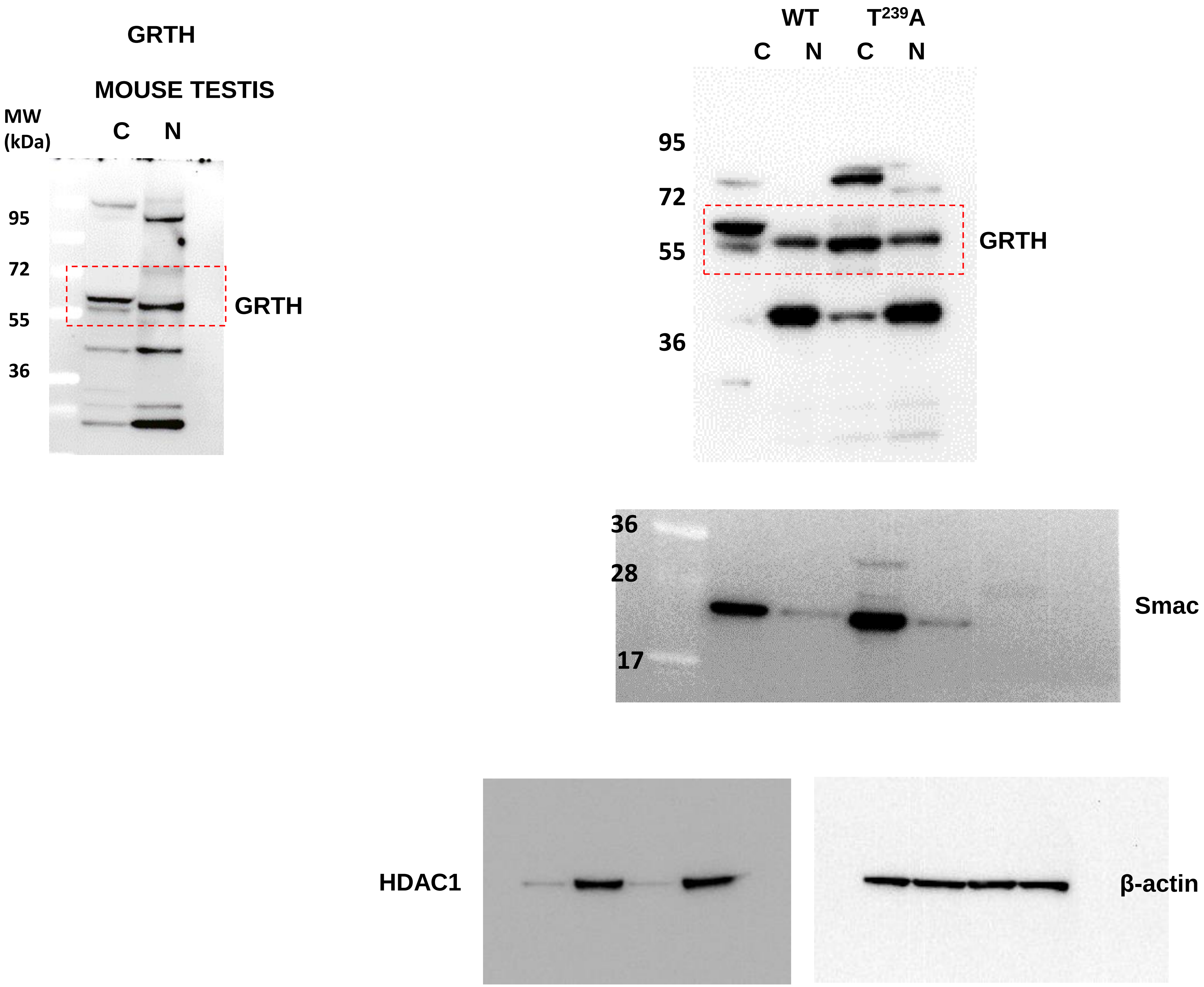
GRTH
MOUSE TESTIS
MW (kDa)
C
N
95
72
55
36
GRTH
WT
T239A
C
N
C
N
95
72
55
36
GRTH
36
28
17
Smac
HDAC1
β-actin

Fig. S2

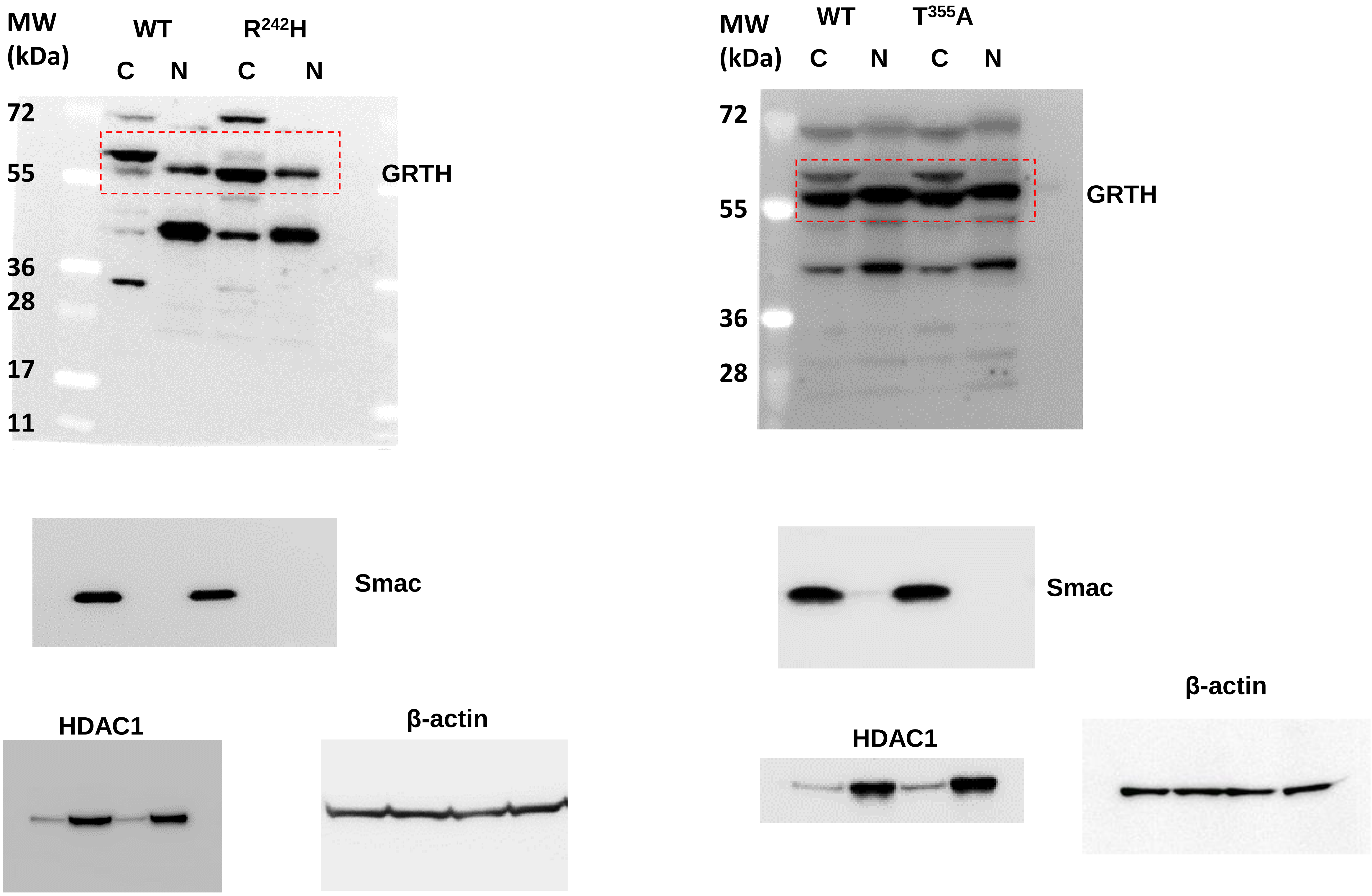

MW (kDa)
WT
R242H
C
N
C
N
72
55
36
28
17
11
GRTH
Smac
HDAC1
β-actin
MW (kDa)
WT
T355A
C
N
C
N
72
55
36
28
GRTH
Smac
HDAC1
β-actin

Fig. S3

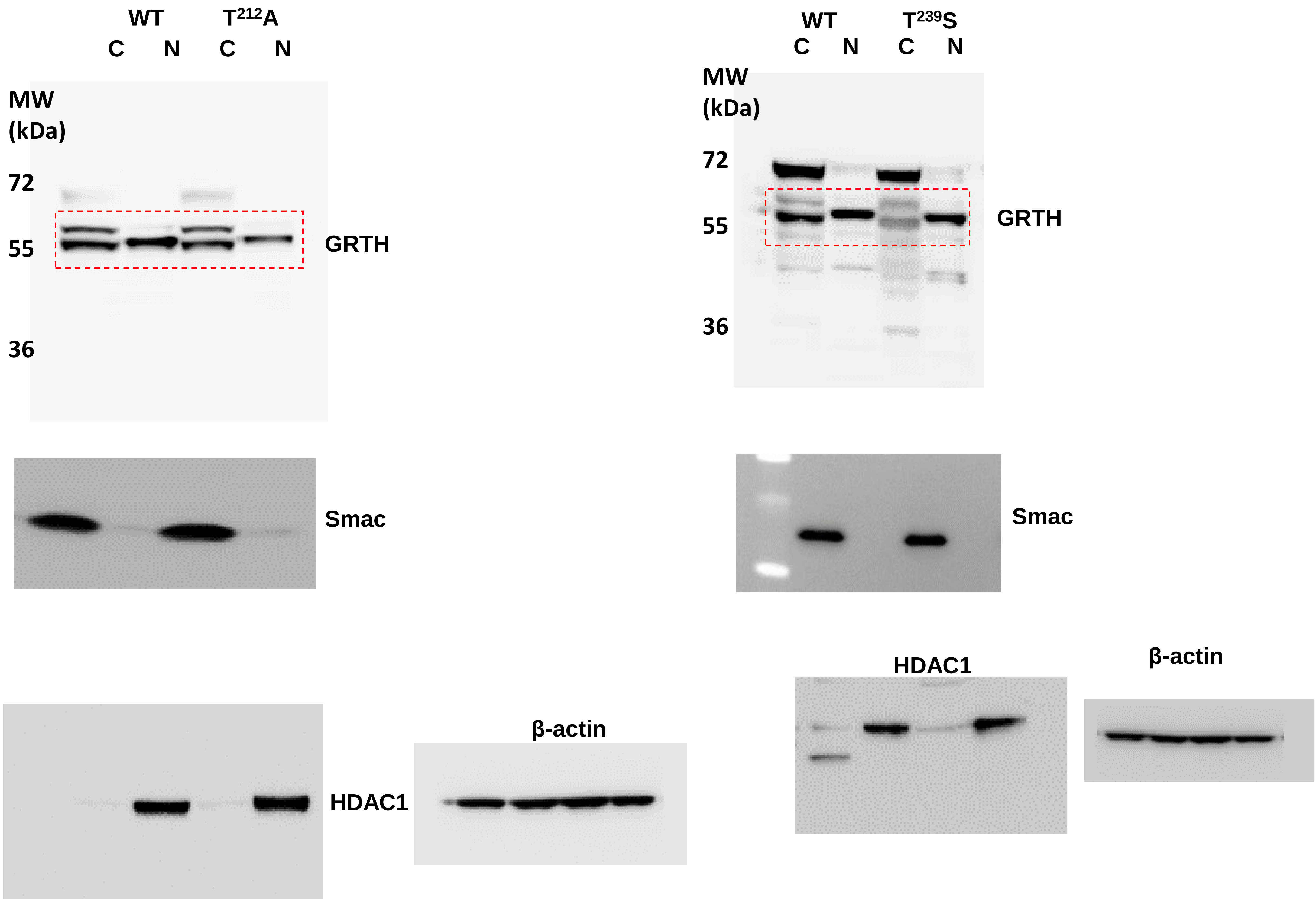
WT
T212A
C
N
C
N
MW
(kDa)
72
55
36
GRTH
Smac
HDAC1
β-actin
WT
T239S
C
N
C
N
MW
(kDa)
72
55
36
GRTH
Smac
HDAC1
β-actin

Fig. S4

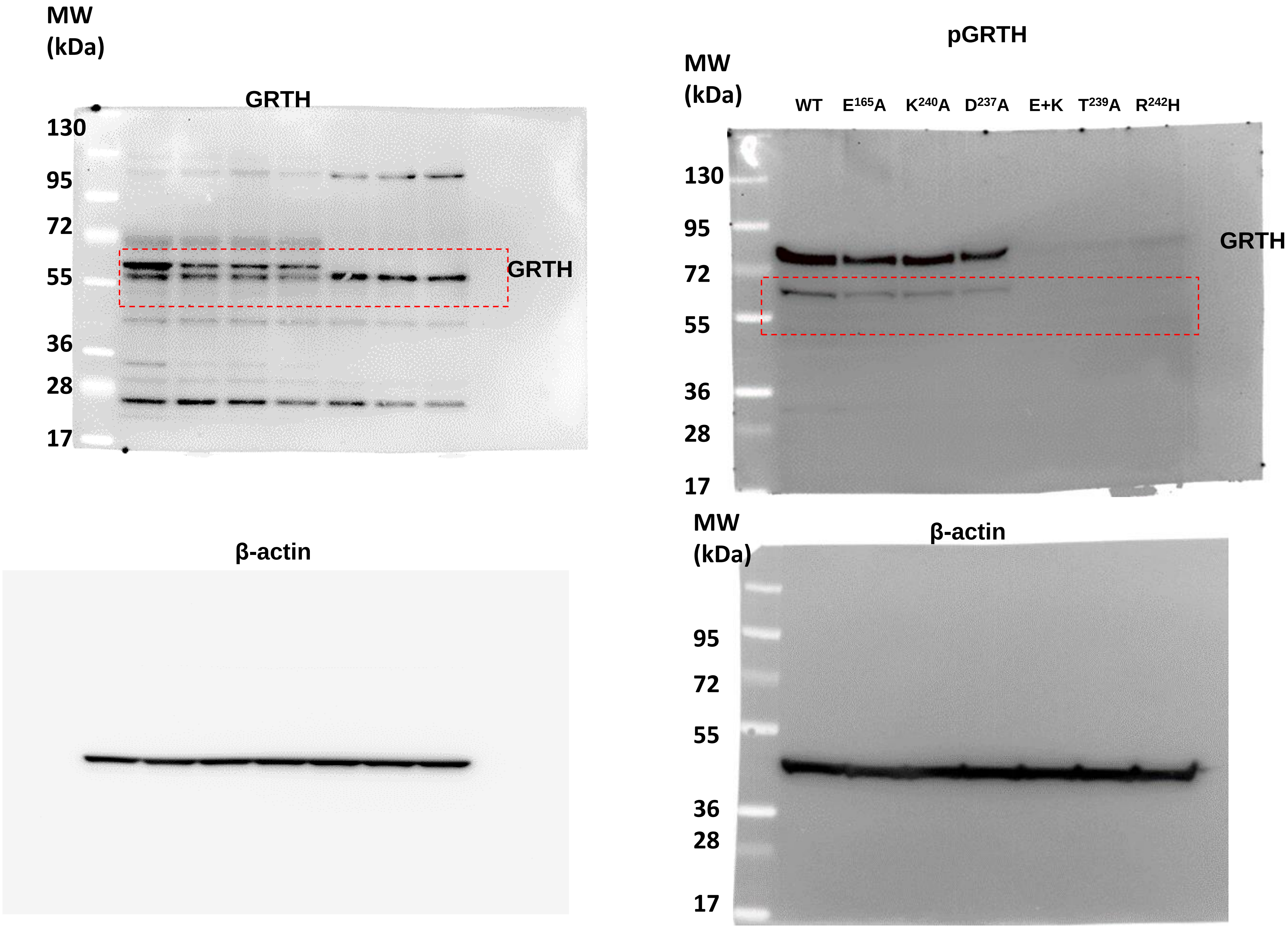
MW
(kDa)
GRTH
130
95
72
55
36
28
17
GRTH
β-actin
pGRTH
MW
(kDa)
WT
E165A
K240A
D237A
E+K
T239A
R242H
130
95
72
55
36
28
17
GRTH
MW
(kDa)
β-actin
95
72
55
36
28
17

Fig. S5

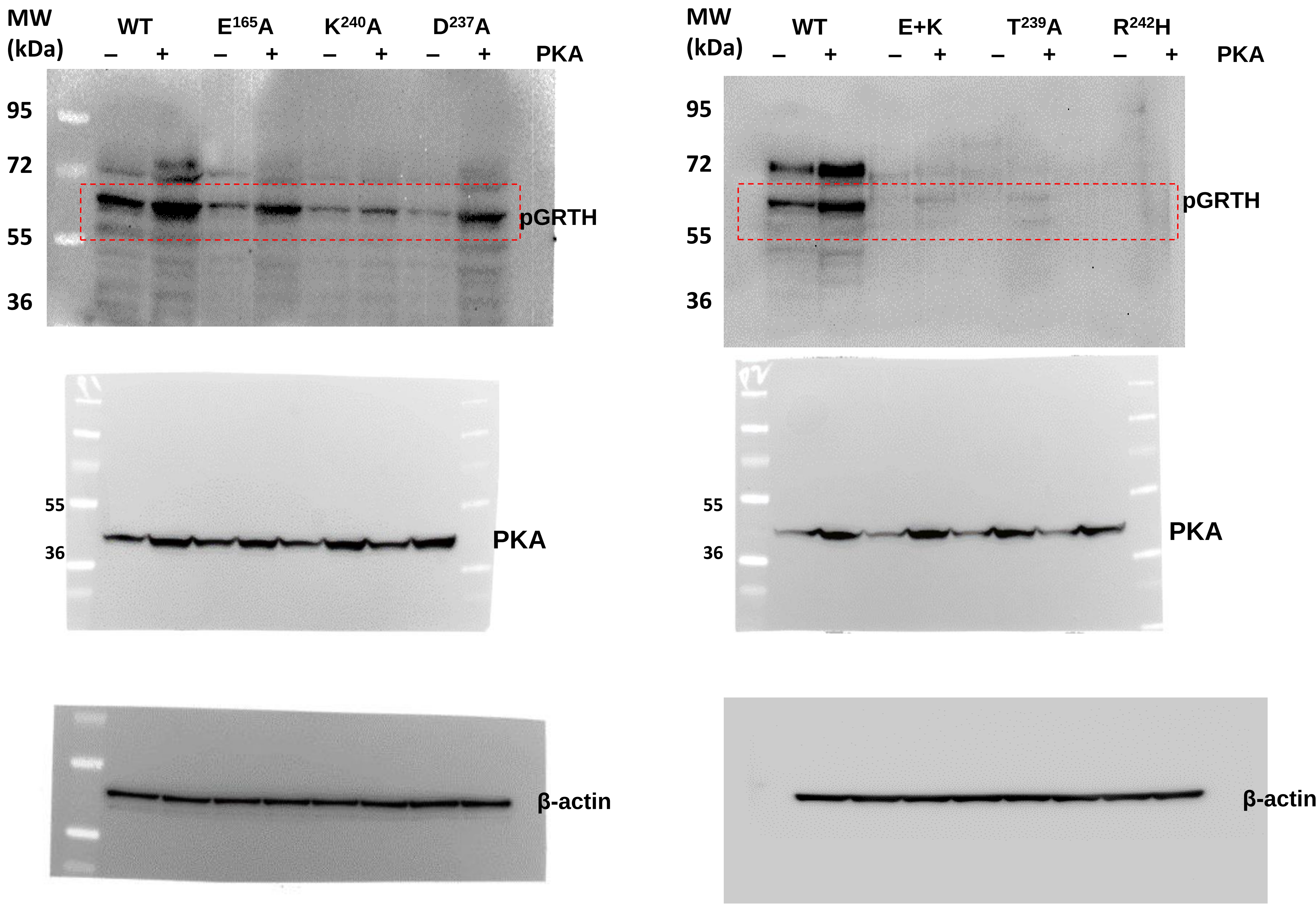
MW (kDa)
WT
E165A
K240A
D237A
– + – + – + – + PKA
95
72
55
36
pGRTH
55
36
PKA
β-actin
MW (kDa)
WT
E+K
T239A
R242H
– + – + – + – + PKA
95
72
55
36
pGRTH
55
36
PKA
β-actin

Fig. S6

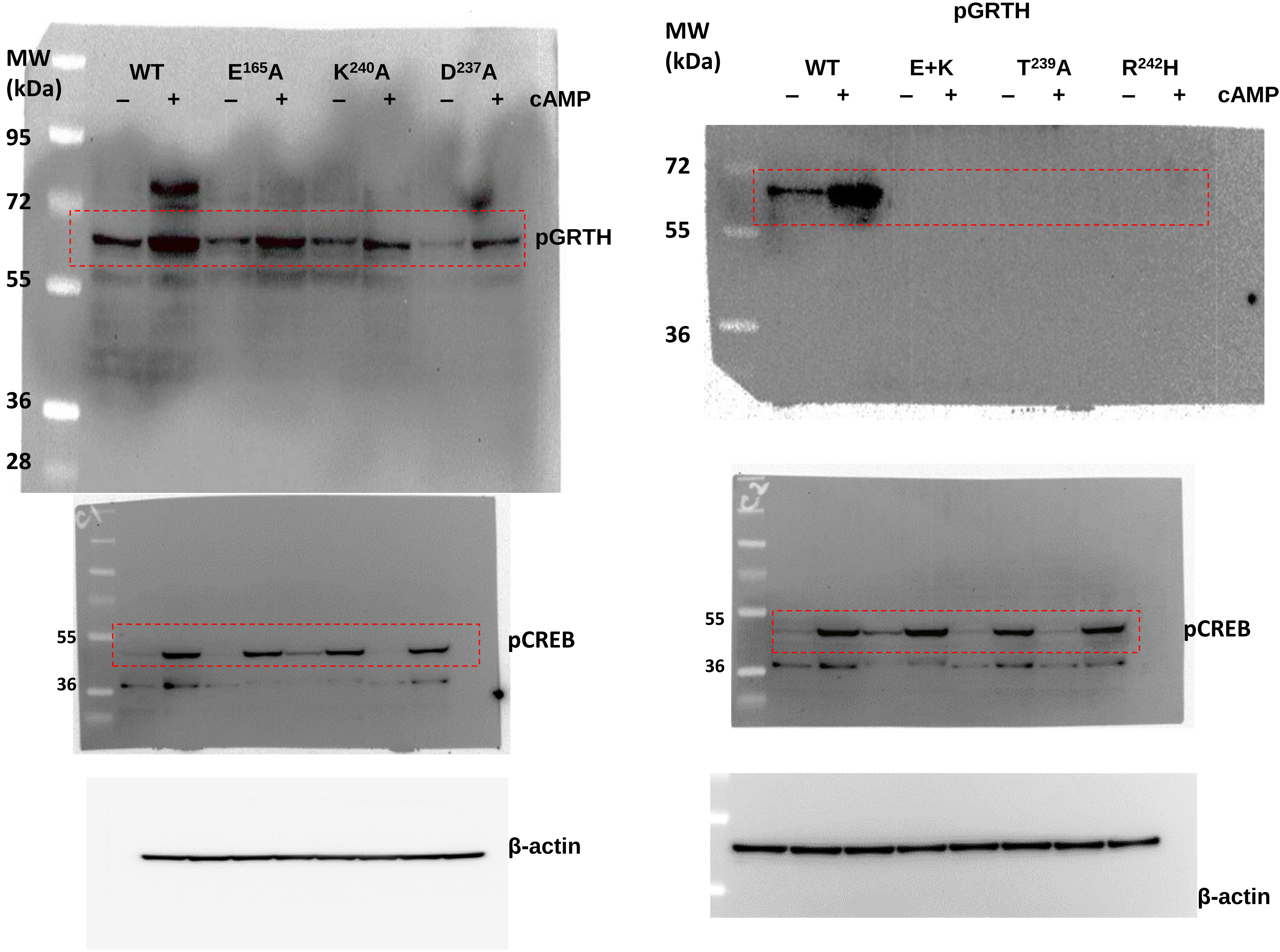

MW (kDa)
WT
E165A
K240A
D237A
– + – + – + – +
cAMP
95
72
55
36
28
pGRTH
pCREB
55
36
β-actin
pGRTH
MW (kDa)
WT
E+K
T239A
R242H
– + – + – + – +
cAMP
72
55
36
pCREB
55
36
β-actin

Fig. s7

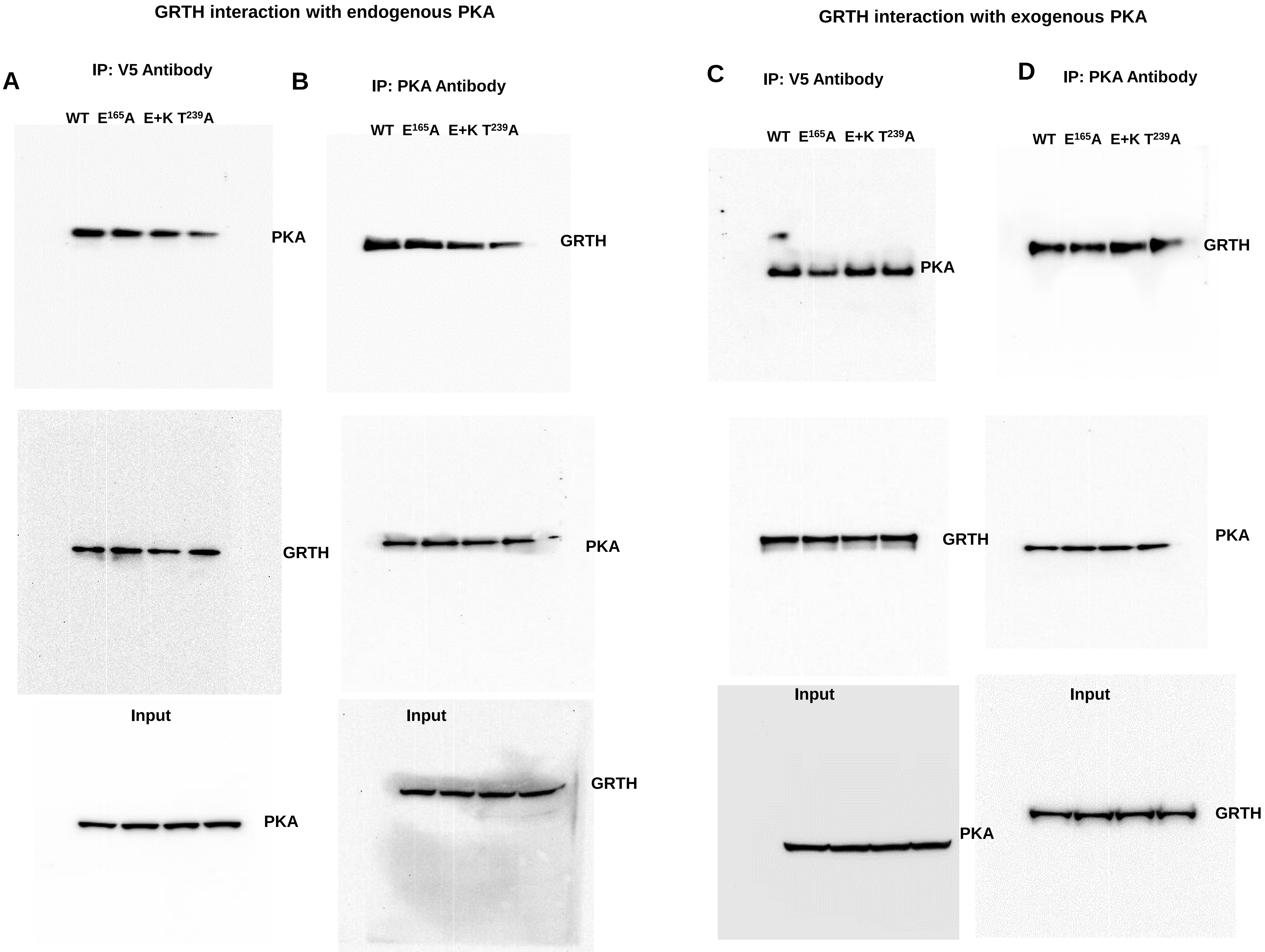
GRTH interaction with endogenous PKA
GRTH interaction with exogenous PKA
A
IP: V5 Antibody
WT E165A E+K T239A
PKA
GRTH
Input
PKA
B
IP: PKA Antibody
WT E165A E+K T239A
GRTH
PKA
Input
GRTH
C
IP: V5 Antibody
WT E165A E+K T239A
PKA
GRTH
Input
PKA
D
IP: PKA Antibody
WT E165A E+K T239A
GRTH
PKA
Input
GRTH
